# Childhood Obesity: Issues of Weight Bias

**Published:** 2011-08-15

**Authors:** Reginald L. Washington

**Affiliations:** Rocky Mountain Hospital for Children. Dr Washington is also affiliated with the University of Colorado School of Medicine

## Abstract

Although the effects of obesity on children's physical health are well documented, the social consequences of obesity are less well described and may not be addressed in intervention programs. Weight bias may take several forms. It may result in teasing and discrimination and may affect employment and educational opportunities. Health care providers may limit care of overweight or obese children. The media promote weight bias in multiple ways. Some parents are biased against their obese children. In an effort to avoid weight bias, new efforts to reduce obesity must be evaluated to determine whether these efforts do, in fact, add to the problem. It is important to understand that the weight bias that obese youth face is just as serious as the physical consequences of excessive weight on the welfare of the child.

## Introduction

The obesity epidemic continues in the United States. Although the effects of obesity on the physical health of children are well documented, the emotional and social consequences of obesity are less detailed and not as well understood, and therefore are often ignored. The emotional consequences of obesity include low self-esteem, negative body image, and clinical depression (1). Any action or policy that exacerbates these consequences is considered a serious ethical problem. Obesity affects social health as well. These social effects often take the form of weight bias or stigma. This article summarizes what is known about weight bias based on selected reports. It is not intended to be a review of the literature. I hope that the opinions offered will increase interest in weight bias and its effects on children and generate further discussion.

## Weight Bias

*Weight bias* can be defined as the inclination to form unreasonable judgments based on a person's weight. *Stigma* is the social sign that is carried by a person who is a victim of prejudice and weight bias. Obese children are at an increased risk for bias as a result of their weight.

Weight bias is caused by a general belief that stigma and shame will motivate people to lose weight or the belief that people fail to lose weight as a result of inadequate self-discipline or insufficient willpower. Our culture may not punish people who practice weight bias because our culture values thinness (1). Society frequently blames the victim rather than addressing environmental conditions that contribute to obesity.

Weight bias affects the child in multiple ways. Obese children are often the brunt of teasing or discrimination. Bias exists in the adult workplace and may affect children as they enter the workforce. Weight bias also influences educational success and may affect how health care is delivered. Weight bias is promoted in the media and even by parents of obese children. Curbing the obesity epidemic will require new strategies that do not result in bias or prejudice.

## Weight Bias and Teasing

Teasing, a potential problem for all youth, is especially a problem for overweight and obese youth regardless of racial or ethnic group. In a study by van den Berg et al, one-quarter to one-half of children teased by family or peers were bothered by it, and more white females were disturbed than were people in other groups (2). Asian American adolescents may experience somewhat less weight-biased teasing from peers and more weight-biased teasing from family members. Efforts to eradicate weight bias can provide benefits to a sizeable number of adolescents across different racial/ethnic groups (2).

## Weight Bias and Discrimination

Perceived weight and height discrimination have increased from 1995-1996 to 2004-2006, from 7% to 12% (3). During this same period, perceived racial/ethnic discrimination has remained stable, and the prevalence of weight and height discrimination has increased to levels that are now comparable with those reported for race and age discrimination (3).

Three hypotheses may explain the increase in weight discrimination. First, rates of obesity have escalated during the same period. Second, perceived weight discrimination may reflect experiences that have resulted from worsening societal attitudes and the acceptance of weight bias. Third, the media contribute and encourage weight bias and discrimination.

Information that emphasizes personal responsibility as the principal cause of obesity worsens negative stereotypes and increases bias toward obese people (4). Obese people are more likely to be blamed and negatively stereotyped when they are perceived by others to be personally responsible for their weight gain; however, they receive more favorable evaluations and less blame when obesity can be attributed to a physical cause outside of their personal control, especially among children (5).

## Weight Bias and Employment

Weight bias against obese adults in the workplace has been widely reported (6,7). Although studies involving children in the workplace do not exist, obese children are at great risk of becoming obese adults and, therefore, are at risk for bias in the workplace. Compared with job applicants who have similar qualifications, obese applicants are rated more negatively and are less likely to be hired. Obese applicants are also perceived to be unfit for jobs involving face-to-face interactions. Overweight and obese applicants are viewed as having inadequate self-discipline, low supervisory potential, unacceptable personal hygiene, and less ambition and productivity (6). As a consequence, overweight and obese employees earn 1% to 6% less than normal-weight people in comparable positions, and this salary difference is greater for obese women than obese men (7).

Obese people are promoted less often and are sometimes viewed by their employers and coworkers as lazy, less competent than their peers, and lacking self-discipline (8). Obese people believe they can be fired or suspended because of their weight, despite demonstrating satisfactory job performance, even when weight is unrelated to their job responsibilities (9).

## Weight Bias and Education

Teachers perceive overweight students to be untidy, overly emotional, less likely to succeed at work, and more likely to have family problems compared with their normal-weight peers (10). Forty-three percent of principalsagree that most people are uncomfortable when they associate with obese people (11). In addition, teachers have lower expectations for overweight students compared with thinner students across a range of ability areas (12). Obese students are less likely to be accepted for admission into college, despite having comparable academic performance (13). These facts may explain why obese students feel a bias during their educational experiences.

Approximately 1 in 3 overweight females and 1 in 4 overweight males report being teased by peers at school; among the heaviest group of youth, that figure increases to 3 in 5 (1). Peers regard obese children as undesirable playmates who are lazy, stupid, ugly, mean, and unhappy (1). Negative attitudes begin in preschool and can worsen as children age (14). As a consequence, obese elementary school children miss more days of school than do their nonobese peers (15). Obese adolescent females are less likely to attend college than are nonobese females (16). Students who were obese at age 16 years had fewer years of education compared with nonobese peers, and youth who are victimized because of their weight are more vulnerable to depression, low self-esteem, inferior body image, and suicidal thoughts (17). Weight-biased teasing makes the youngster more likely to engage in unhealthful eating habits and to avoid physical activity in school (1).

## Weight Bias and Health Care Discrimination

In a study of 400 doctors, 1 in 3 listed obesity as a condition to which they responded negatively. Doctors ranked obesity right behind drug addiction, alcoholism, and mental illness. They also associated obesity with noncompliance, hostility, dishonesty, and inadequate hygiene (18). Consequently, overweight patients may be reluctant to seek medical care, may cancel or delay medical appointments, or may postpone seeking important preventive services (19). Doctors examining overweight patients spend less time with them, engage in less discussion, are reluctant to perform preventive health screenings, and do fewer interventions (20). Self-monitoring — teaching the patient in a positive way to learn to monitor diet and activity to achieve weight maintenance or gradual weight loss — should be distinguished from making overweight and obese children feel guilty about personal habits related to weight gain. Educational efforts should be directed to health care providers to make them aware of the potential negative role they may play by giving negative messages.

## Weight Bias and the Media

Children's media have a prevailing tendency to represent positive messages about being thin and negative messages about being overweight. In children's entertainment, thin characters are ascribed desirable attributes and dominate central roles (21), whereas overweight characters are onscreen rarely or in minor stereotypical roles. Compared with thin characters appearing on television, heavier characters rarely are portrayed in romantic relationships (and never with thin characters), are more likely to be objects of humor and ridicule, and often engage in stereotypical eating behaviors (22).

## Weight Bias and Parents

In a study of 5-year-old girls, parents who were overweight (60% of mothers and 82% of fathers) or obese (28% of mothers and 31% of fathers) were just as likely to endorse negative stereotypes as were thinner parents (23). Girls were more likely than boys to display negative stereotypes if their parents emphasize the importance of thin body shape and weight loss. Fathers with higher education and income were more likely to endorse stereotypes than were fathers with less education and income, as were parents who reported a strong investment in their own appearance compared with parents who did not (23).

In 1 study, weight-biased teasing by family members was reported by 47% of substantially overweight females and 34% of substantially overweight males (1). Bias from parents may have unexpected consequences. Studies among high school seniors have demonstrated that overweight females receive less financial support from their parents for college than do average-weight females, even after controlling for parental income, race/ethnicity, family size, and education (24).

## Weight Bias, Prevention, and Eating Disorders

As increased attention is paid to the obesity epidemic it would be unethical to, at the same time, increase weight bias. Efforts to promote environmental and policy changes to decrease overeating and inactivity (including banning competitive foods from schools, mandating menu labeling, reporting students' body mass index to parents, and requiring daily physical education) are met with concern that these activities may lead to an increase in societal preoccupation with dietary restraint and worsening body image, thereby increasing weight bias and the incidence of eating disorders (25). Data demonstrate, however, that recent societal focus on obesity prevention has not led to a discernible increase in eating and eating disorder behavior, and no evidence exists that increased media or professional discussions regarding childhood obesity have been associated with a concomitant increase in eating pathologies (25).

## Conclusion

The obesity epidemic continues to spread at an alarming rate. Preventing childhood obesity has become a priority in an effort to improve the nation's health. The reduction of weight bias is just as important as the reduction of body mass index is. Children need adults to advocate for them and to fight against weight bias, especially as new interventions are developed. Although the effects of weight bias are not as well known, the consequences are just as serious as excessive weight is on the welfare of the child. Future studies are needed to better understand bias, especially the effect it has on the education and employment of children. Additional research is needed to determine how best to educate teachers and health care providers so that they do not add bias as they interact with obese children.

## References

[1] Puhl RM, Latner JD (2007). Stigma, obesity, and the health of the nation's children. Psychol Bull.

[2] van den Berg P, Neumark-Sztainer D, Eisenberg ME, Haines J (2008). Racial/ethnic differences in weight-related teasing in adolescents. Obesity (Silver Spring).

[3] Andreyeva T, Puhl RM, Brownell KD (2008). Changes in perceived weight discrimination among Americans, 1995-1996 through 2004-2006. Obesity (Silver Spring).

[4] Puhl RM, Schwartz MB, Brownell KD (2005). Impact of perceived consensus on stereotypes about obese people: a new approach for reducing bias. Health Psychol.

[5] Anesbury T, Tiggemann M (2000). An attempt to reduce negative stereotyping of obesity in children by changing controllability beliefs. Health Educ Res.

[6] Pingitore R, Dugoni BL, Tindale RS, Spring B (1994). Bias against overweight job applicants in a simulated employment interview. J Appl Psychol.

[7] Baum CL, Ford WF (2004). The wage effects of obesity: a longitudinal study. Health Econ.

[8] Roehling MV (1999). Weight-based discrimination in employment: psychological and legal aspects. Personnel Psychology.

[9] Rothblum ED, Brand PA, Miller CT, Oetjen HA (1990). The relationship between obesity, employment discrimination, and employment-related victimization. J Vocat Behav.

[10] Neumark-Sztainer D, Story M, Harris T (1999). Beliefs and attitudes about obesity among teachers and school health care providers working with adolescents. J Nutr Educ.

[11] Price JH, Desmond SM, Stelzer CM (1987). Elementary school principals' perceptions of childhood obesity. J Sch Health.

[12] O'Brien KS, Hunter JA, Banks M (2007). Implicit anti-fat bias in physical educators: physical attributes, ideology and socialization. Int J Obes (Lond).

[13] Canning H, Mayer J (1966). Obesity — its possible effect on college acceptance. N Engl J Med.

[14] Eisenberg ME, Neumark-Sztainer D, Story M (2003). Associations of weight-based teasing and emotional well-being among adolescents. Arch Pediatr Adolesc Med.

[15] Geier AB, Foster GD, Womble LG, McLaughlin J, Borradaile KE, Nachmani J (2007). The relationship between relative weight and school attendance among elementary schoolchildren. Obesity (Silver Spring).

[16] Needham BL, Crosnoe R, Muller C (2004). Academic failure in secondary school: the inter-related role of health problems and educational context. Soc Probl.

[17] Sargent JD, Blanchflower DG (1994). Obesity and stature in adolescence and earnings in young adulthood. Analysis of a British birth cohort.. Arch Pediatr Adolesc Med.

[18] Klein D, Najman J, Kohrman AF, Munro C (1982). Patient characteristics that elicit negative responses from family physicians. J Fam Pract.

[19] Olson CL, Schumaker HD, Yawn BP (1994). Overweight women delay medical care. Arch Fam Med.

[20] Bertakis KD, Azari R (2005). The impact of obesity on primary care visits. Obes Res.

[21] Herbozo S, Tantleff-Dunn S, Gokee-Larose J, Thompson JK (2004). Beauty and thinness messages in children's media: a content analysis. Eat Disord.

[22] Greenberg BS, Eastin M, Hofschire L, Lachlan K, Brownell KD (2003). Portrayals of overweight and obese individuals on commercial television. Am J Public Health.

[23] Davison KK, Birch LL (2001). Weight status, parent reaction, and self-concept in five-year-old girls. Pediatrics.

[24] Crandall CS (1995). Do parents discriminate against their heavyweight daughters?. Pers Soc Psychol Bull.

[25] Schwartz MB, Henderson KE (2009). Does obesity prevention cause eating disorders?. J Am Acad Child Adolesc Psychiatry.

